# Targeting Polyploid Giant Cancer Cells (PGCCs) to Prevent Cancer Recurrence: Opportunities for Nanomedicine

**DOI:** 10.3390/cancers18203411

**Published:** 2026-10-21

**Authors:** Jillian Norton, Youssef W. Naguib

**Affiliations:** School of Pharmacy, Pacific University, Hillsboro, OR 97123, USA; nort9109@pacificu.edu

**Keywords:** polyploid giant cancer cells, therapy tolerance, ferroptosis, PGCC, nanomedicine, cancer recurrence, stress adaptation, cancer resistance

## Abstract

**Background/Hypothesis:** Therapeutic resistance and recurrence remain major barriers to durable cancer control because conventional therapies may eliminate proliferating tumor cells while allowing dormant, stem-like, senescence-like, or polyploid populations to persist. This review examines polyploid giant cancer cells (PGCCs) as therapy-tolerant states and evaluates whether their adaptive survival mechanisms create vulnerabilities that could be targeted through ferroptosis-oriented nanomedicine. **Methods:** We investigated recent evidence (PubMed) linking PGCC persistence with redox buffering, glutathione/GPX4 activity, iron regulation, lipid remodeling, autophagy, mitochondrial adaptation, and metabolic plasticity, in addition to nanomedicine potential to target these pathways. **Results and Findings:** Emerging studies provide direct proof-of-concept that PGCCs can exhibit ferroptosis susceptibility, while nanomedicine offers potential strategies to improve therapeutic localization and coordinate iron delivery, reactive oxygen species amplification, glutathione depletion, GPX4 inhibition, and combination therapy. However, PGCC-selective nanoparticle efficacy remains largely untested, and important barriers include heterogeneous tumor penetration, off-target toxicity, model limitations, and biomarker uncertainty. **Conclusions and Perspective:** The specific contribution of this review is to integrate PGCC biology, therapy-tolerant cancer states, ferroptosis, and nanomedicine into a unified framework for targeting residual disease. This perspective is timely as direct evidence of PGCC ferroptosis sensitivity and new characterization of treatment-resistant PGCC states are emerging. By identifying the mechanistic overlap among these fields, this review highlights testable opportunities for eliminating persistent cancer cells that may otherwise contribute to recurrence.

## Simple Summary

Cancer can return after treatment because some cancer cells survive chemotherapy, radiation, and other forms of stress. One group of surviving cells, called polyploid giant cancer cells (PGCCs), can become very large, remain inactive for long periods, and later produce new cancer cells that may contribute to tumor regrowth and treatment resistance. This review examines how these cells survive hostile tumor conditions and considers whether their dependence on processes such as antioxidant defenses, iron regulation, lipid metabolism, and cellular recycling could create new treatment opportunities. In particular, we discuss ferroptosis, a form of cell death caused by iron-dependent damage to cell membranes, and the potential use of nanomedicine to deliver therapies that trigger this process. Better understanding these stress-adapted cancer cells may help researchers develop treatments aimed at eliminating residual cancer cells and reducing the risk of cancer recurrence.

## 1. Introduction

One of the toughest challenges in modern oncology is the persistent failure of therapies to produce durable responses, resulting in recurrence and resistance across nearly all cancer types. This limitation is particularly evident in malignancies such as glioblastoma, pancreatic ductal adenocarcinoma (PDAC), and triple-negative breast cancer (TNBC), where initial responses to therapy are frequently followed by relapse and disease progression despite aggressive treatment regimens [1,2,3,4,5]. These outcomes reflect not only cell-intrinsic resistance mechanisms, but also the persistence of adaptive cancer cell states and tumor-level barriers that prevent complete therapeutic eradication [4,5,6,7].

Traditionally, cancer treatment paradigms have been built on a binary framework in which cells either survive or undergo regulated cell death pathways such as apoptosis. However, this model is increasingly insufficient to explain clinical outcomes. As highlighted by Mirzayans and Murray (2023), conventional definitions of “cell death” and “lethality” are often misleading, as many cancer cells classified as non-viable based on proliferative assays remain metabolically active and persist following treatment [6]. Experimental observations demonstrate that treatment-exposed cancer cells can enter enlarged, non-proliferative states that fail clonogenic assays yet remain viable for extended periods and retain the capacity to influence tumor progression [6,8]. Together, these findings suggest that current frameworks for evaluating therapeutic success may underestimate the persistence of biologically active, therapy-resistant cell populations.

Conventional chemotherapy faces several barriers that extend beyond the intrinsic sensitivity of cancer cells. Multidrug resistance can arise through increased drug efflux, impaired apoptotic signaling, altered drug targets, enhanced DNA repair, and metabolic adaptation, while solid tumors further restrict therapeutic effectiveness through abnormal vasculature, elevated interstitial fluid pressure, hypoxia, acidic extracellular conditions, and heterogeneous drug penetration [9,10,11]. Nanomedicine provides a means of addressing several of these limitations by modifying drug pharmacokinetics and biodistribution, improving the solubility and stability of therapeutic agents, increasing cellular uptake, and enabling controlled or combination delivery [9,10,11]. Nanocarriers including liposomes, polymeric nanoparticles, micelles, dendrimers, and inorganic systems can also be engineered for passive or active tumor targeting and stimulus-responsive release, creating opportunities to address both cellular and microenvironmental mechanisms of chemotherapy resistance [9,10,11].

Nanomedicine may be particularly relevant to residual tumor populations that are poorly addressed by proliferation-dependent chemotherapy. Cancer stem cells (CSCs), for example, can exhibit quiescence, enhanced drug-efflux activity, metabolic plasticity, and resistance to conventional cytotoxic therapy [9,12,13]. Nanomedicine-based approaches have therefore been investigated to deliver chemotherapeutic agents or nucleic acids to CSCs, inhibit drug-efflux mechanisms, target stemness-associated signaling or metabolism, and exploit cell-surface markers to improve selective uptake [9,12,13]. Although PGCCs are biologically distinct from CSCs, their shared features of treatment persistence, stem-like behavior, metabolic adaptation, and capacity for tumor repopulation make these established nanomedicine principles relevant to the development of PGCC-directed strategies.

Within this framework, this review examines PGCCs as a therapy-tolerant cellular state and evaluates whether the adaptive dependencies that support their persistence, including altered redox regulation, lipid metabolism, iron homeostasis, mitochondrial function, and autophagy, may create therapeutic opportunities for ferroptosis-oriented nanomedicine. Because direct evidence for PGCC-selective nanoparticle therapy remains limited, findings from broader therapy-tolerant and stem-like cancer populations are distinguished from evidence demonstrated specifically in PGCCs.

Recent literature has separately examined PGCC biology, ferroptosis, cancer stem-cell-directed nanomedicine, and nanoparticle strategies for treatment-resistant disease. However, these topics have largely been evaluated as distinct areas of investigation. Table 1 summarizes representative recent studies and reviews and highlights the gap addressed by the present review, which integrates PGCC-associated therapy tolerance with ferroptosis-relevant vulnerabilities and nanomedicine-based delivery strategies.

Collectively, these studies establish strong individual foundations in PGCC biology, therapy resistance, ferroptosis, and nanomedicine. The present review extends this literature by linking these fields into a unified framework focused specifically on residual PGCC-like states and the potential use of ferroptosis-oriented nanomedicine to disrupt the adaptive mechanisms that permit their persistence.

## 2. Therapy Resistance and Tumor Heterogeneity

### 2.1. Therapy-Tolerant Cellular States Contribute to Resistance

A critical limitation of current therapeutic strategies is the assumption that non-proliferating cells are biologically nonviable. However, increasing evidence demonstrates that cancer cells can enter therapy-tolerant states, including dormancy, quiescence, and senescence-like phenotypes, in response to treatment-induced stress [5,6,7]. These states represent adaptive survival programs that allow cells to persist under conditions such as hypoxia, nutrient deprivation, and cytotoxic exposure, and have been documented across multiple tumor types, including breast cancer, where dormant tumor cells can survive for extended periods before reactivation and metastatic recurrence [3,7].

Importantly, therapy-tolerant cells are not biologically inert. Persistent cells can actively influence the tumor microenvironment (TME) through secretion of cytokines, growth factors, and extracellular vesicles, consistent with the senescence-associated secretory phenotype (SASP), which promotes tumor progression, immune modulation, and resistance [6,7,21]. In parallel, these cells may continue to undergo genomic and epigenetic alterations that enhance their adaptive potential under therapeutic pressure [5,6].

A related and well-established concept that further supports the persistence of therapy-tolerant populations is the existence of cancer stem cells (CSCs), a subpopulation of tumor cells characterized by self-renewal capacity, differentiation potential, and intrinsic resistance to conventional therapies [22,23]. Cancer stem cells have been identified across multiple malignancies, including breast cancer, glioblastoma, and pancreatic cancer, where they contribute to tumor initiation, metastasis, and relapse following treatment [22,23]. Significantly, CSCs are often enriched following cytotoxic therapy, suggesting that standard treatments may preferentially eliminate proliferating cells while sparing or promoting stem-like populations. Mechanistically, CSCs exhibit multiple resistance-promoting features, including enhanced DNA damage repair, activation of pro-survival signaling pathways, metabolic flexibility, and the ability to exist in quiescent or slow-cycling states, all of which contribute to their capacity to evade therapeutic elimination and drive long-term disease recurrence [22,23].

Emerging evidence suggests that polyploid giant cancer cells (PGCCs), enlarged cancer cells characterized by a single polyploid nucleus or multiple nuclei, share several functional and phenotypic characteristics with CSCs, particularly their capacity to survive therapeutic stress and exhibit stem-like properties [7,8]. During exposure to chemotherapy or radiation, PGCCs may persist in a non-proliferative, therapy-tolerant state [7]. Following the cessation of treatment and removal of therapeutic stress, surviving PGCCs can undergo depolyploidization, generating daughter cells with renewed proliferative, migratory, and invasive behavior [7]. Therefore, in this context, PGCCs exhibit stem-like characteristics, whereas their daughter cells contribute to tumor repopulation through renewed proliferation and invasive behavior. This process effectively links therapy-induced stress responses to cellular reprogramming and tumor regeneration, suggesting that PGCCs may function as transient reservoirs for residual disease rather than terminally damaged or biologically irrelevant cells [7,8].

For the purposes of this review, PGCC identification should not be based on cell size or multinucleation alone. Therapy-induced enlargement and multinucleation may also occur in cells undergoing irreversible senescence, mitotic catastrophe, or terminal injury. Stronger evidence for a functionally relevant PGCC state includes continued viability after treatment, persistence following withdrawal of the inducing stress, and, most importantly, demonstration of depolyploidization or noncanonical division yielding viable proliferative progeny [6,7,8,18,24]. Because the studies discussed in this review vary in the extent to which these functional criteria were evaluated, morphological identification of polyploid or multinucleated giant cells is distinguished from direct evidence of reproductive competence wherever possible.

Within this broader landscape of therapy tolerance, PGCCs should be introduced as one example of a larger adaptive survival framework rather than as an isolated phenomenon. Dormancy, quiescence, senescence-like persistence, cancer stem-like behavior, epithelial–mesenchymal transition (EMT)-associated plasticity, and polyploidization all represent overlapping responses to therapeutic and microenvironmental stress [5,6,7,8,18,24,25,26,27]. The significance of PGCCs lies in their ability to connect several of these programs at once: they are enlarged and polyploid, frequently stress-resistant, capable of entering prolonged non-proliferative states, and able, in some contexts, to generate progeny with renewed proliferative and tumorigenic capacity. This makes PGCCs particularly relevant to the central problem of this review: residual cancer cells may survive not because therapy has no effect, but because therapy itself can select for adaptive states that remain biologically active after apparent treatment response.

### 2.2. Intratumoral Heterogeneity Drives Resistance

In parallel, intratumoral heterogeneity further complicates treatment outcomes by ensuring that tumors are composed of diverse subpopulations with distinct molecular and phenotypic profiles. As described by Dagogo-Jack and Shaw (2018), this heterogeneity provides the foundation for resistance, allowing pre-existing or evolving subclones to survive therapeutic pressure and expand over time [5].

This process reflects fundamental principles of Darwinian selection, in which therapeutic intervention serves as a selective bottleneck that shapes tumor composition over time [5]. Importantly, resistance does not arise solely from fixed genetic mutations, but also from dynamic and reversible adaptations that enable cells to transition into survival states under stress [6,7]. As a result, intratumoral heterogeneity should be viewed not as a static characteristic, but as an evolving system that continuously generates and selects for cell populations capable of withstanding therapeutic pressure. Particularly, these evolutionary dynamics do not act on genetically distinct populations alone, but also on therapy-induced survival states such as polyploid and stem-like cells, further reinforcing the persistence of resistant populations under continued therapeutic pressure [5,6,7,24,28,29].

### 2.3. Limitations of Current Therapeutic Frameworks

Emerging work in the field is beginning to reconsider these assumptions, with increasing attention directed toward understanding and targeting the survival mechanisms that enable cancer cells to endure therapeutic stress. In particular, there is growing interest in identifying alternative vulnerabilities associated with non-proliferative and therapy-induced states, including metabolic dependencies and stress-response pathways that may be distinct from those of actively dividing cells [7,30].

While these approaches remain under active investigation, they highlight a broader shift toward more integrative models of therapeutic response, ones that account for cellular dormancy, plasticity, and evolutionary dynamics. This shift is especially important for understanding PGCCs, because these cells challenge the assumption that enlarged, non-proliferative, or morphologically abnormal cells are necessarily irrelevant to recurrence. Instead, PGCCs may represent a stress-adapted reservoir within residual disease, linking therapy-induced injury to later tumor repopulation [6,7,8,18,24,26,27,31].

Together, these concepts provide the biological framework for positioning PGCCs within the broader spectrum of therapy-tolerant cancer cell states. Readers seeking more comprehensive discussions of intratumoral heterogeneity and adaptive resistance are directed to references [4,5,6], while dormancy, PGCC biology, and cancer stem-cell-associated resistance are examined in greater depth in references [7,8,22,23].

## 3. Why Resistant Cancer Cell Populations Are Difficult to Eliminate

The physical, microenvironmental, and metabolic barriers governing therapeutic response have been reviewed extensively. Accordingly, this section does not attempt to address each barrier comprehensively but instead summarizes the interacting pressures most relevant to the emergence and persistence of therapy-tolerant cancer cell populations: spatially heterogeneous drug exposure, microenvironmental selection, and cellular stress adaptation [4,21,30,32,33,34].

### 3.1. Therapy Failure as a Multiscale, Systemic Process

Resistant cancer cell populations represent one of the most challenging obstacles to achieving durable therapeutic responses, yet their persistence cannot be understood as a failure of individual cellular mechanisms alone. Rather, the difficulty in eliminating these resistant populations stems from the fact that cancer therapy failure is fundamentally systemic, arising from interacting constraints that operate across multiple biological and physical scales. Tumors function as complex, heterogeneous systems in which malignant cells coexist with a dynamic microenvironment composed of stromal fibroblasts, immune populations, vasculature, and extracellular matrix, all of which dynamically influence treatment response [4].

At the tumor level, these constraints are coupled rather than independent. Physical transport abnormalities create spatially unequal therapeutic exposure; those exposure gradients intersect with hypoxia, acidosis, nutrient deprivation, and stromal signaling; and the resulting conditions favor cellular programs oriented toward survival rather than proliferation [4,21,30,32,33,34]. Resistance should therefore be understood not only as a property of individual cancer cells, but as an emergent outcome of interacting tumor-level conditions that shape which populations survive therapeutic pressure.

### 3.2. Physical and Transport Barriers to Drug Distribution

The physical architecture of solid tumors imposes fundamental limitations on drug distribution, creating persistent regions of inadequate exposure that directly contribute to therapeutic failures and the emergence of resistant cell populations. As Minchinton and Tannock (2006) comprehensively detail, anticancer drugs must penetrate tumor tissue efficiently and reach all cancer cells at therapeutically sufficient concentrations to exert their effects, yet most anticancer drugs exhibit incomplete distribution within tumor tissue [32]. 

This limitation arises from multiple interrelated structural and physiological abnormalities within the TME. Disorganized and inefficient vasculature reduces vascular density and increases the distance between functional blood vessels and tumor cells, while the absence of effective lymphatic drainage leads to elevated interstitial fluid pressure (IFP) [35]. This elevated IFP fundamentally impairs the movement of both small molecular weight drugs and larger macromolecular therapeutics, including antibodies and other biological agents [32,34,35]. In parallel, tumor blood vessels are structurally abnormal, convoluted, hyperpermeable, and spatially heterogeneous, further disrupting pressure gradients and limiting the efficient delivery of drugs and oxygen throughout the tissue [35].

Beyond vascular abnormalities, the extracellular matrix (ECM) presents an additional physical barrier, as its dense and irregular composition slows molecular movement and creates a maze-like barrier for diffusion [32,34,35]. These combined constraints result in pronounced spatial heterogeneity in drug distribution, where cells located farther from blood vessels experience progressively lower drug concentrations [32,34,35]. This spatial heterogeneity in drug distribution means that even within a single tumor nodule, neighboring cells experience vastly different therapeutic pressures, allowing subpopulations with inherent or acquired resistance mechanisms to proliferate preferentially in poorly perfused regions where drug concentrations fall below therapeutic thresholds [32,34,35].

Compounding these microenvironmental constraints are substantial limitations in nanoparticle delivery. In their analysis of published nanoparticle-delivery studies, Wilhelm and colleagues reported that a median of only 0.7% of the injected nanoparticle dose reached solid tumors [36]. This profound delivery inefficiency means that resistant populations face not only intrinsically favorable microenvironmental conditions supporting their survival, but also exposure to subtherapeutic drug concentrations that further accelerate the selection of increasingly refractory clones [5,29,32,36].

Heterogeneous drug distribution is therefore more than a delivery limitation. By exposing spatially protected populations to subtherapeutic concentrations, it creates distinct levels of therapeutic pressure that may eliminate sensitive cells while permitting stress-adapted populations to persist and evolve [5,25,29,32].

### 3.3. The Tumor Microenvironment as a Selective Landscape

The TME should not be viewed solely as a passive barrier to drug delivery. It also functions as a dynamic and spatially heterogeneous selective landscape in which malignant cells interact with stromal, immune, vascular, and extracellular components. Regional differences in oxygenation, nutrient availability, pH, inflammatory signaling, and growth factors create distinct ecological niches within the same tumor [4,21,33,34]. 

These regional environments create powerful selective pressures that actively shape the emergence and expansion of therapy-resistant cell populations [4,5,21,33,34].

Among these factors, hypoxia represents one of the most influential drivers of selection within tumors. Regions of low oxygen tension arise due to abnormal vasculature and inefficient perfusion, leading to stabilization of hypoxia-inducible factors (HIFs) and activation of transcriptional programs that promote survival, angiogenesis, and metabolic adaptation [21]. Hypoxic conditions have been shown to reduce sensitivity to both chemotherapy and radiation, in part by limiting drug delivery and promoting cellular states that are less dependent on proliferation [21,33,34]. In addition, hypoxia can induce quiescence or slow-cycling behavior, enabling cells to evade therapies that preferentially target rapidly dividing populations [21,33,34]. 

Beyond hypoxia alone, additional microenvironmental stressors, including oxidative stress, nutrient deprivation, and inflammatory signaling, further intensify selective pressure within tumors. Limited nutrient availability can drive shifts towards survival-oriented metabolic states, while inflammatory cues from stromal and immune cells can activate pro-survival signaling pathways that enhance resistance. These overlapping stressors create a compounded selective environment in which only cells capable of adapting to multiple simultaneous constraints can persist, reinforcing the emergence of therapy-resistant populations [4,21,30,33,34].

Physical barriers influence where and at what concentration therapy is delivered, whereas the microenvironment helps determine how cells respond to that exposure. Together, these processes convert spatial heterogeneity into biological selection [4,21,33,34].

### 3.4. Metabolic Reprogramming and Stress Adaptation

In response to the diverse and often hostile conditions imposed by the TME, cancer cells exhibit a high degree of metabolic and adaptive plasticity that enables survival under stress [25]. Rather than passively enduring unfavorable conditions, cancer cells actively reprogram their metabolic pathways to meet the demands of fluctuating oxygen levels, nutrient scarcity, and therapeutic pressures [30]. These adaptations include shifts in glucose metabolism, alterations in mitochondrial function, and increased reliance on alternative energy sources, allowing cells to maintain viability even when classical growth pathways are suppressed [30,37].

Importantly, these adaptive responses extend beyond energy production and involve coordinated regulation of redox homeostasis, lipid metabolism, and cellular stress-response pathways [30,38]. Cancer cells frequently upregulate antioxidant systems to manage increased reactive oxygen species (ROS), while alterations in lipid metabolism support membrane remodeling and survival signaling [30,38]. Autophagy further contributes by enabling the recycling of intracellular components under stress conditions, supporting both metabolic flexibility and long-term persistence [39,40]. These interconnected processes enable cancer cells to enter states that are not only resistant to therapy, but also capable of reactivation once favorable conditions are restored [7,21,25,38,39,40].

Metabolic plasticity should therefore be viewed not simply as a consequence of tumor stress, but as an active survival program that enables cells to remain viable until more favorable conditions permit reactivation [25,30,38].

### 3.5. Convergent Pressures Driving Therapy-Tolerant Cell States

Collectively, therapy-tolerant populations emerge through the convergence of incomplete drug exposure, microenvironmental selection, and cellular adaptation. Physical transport limitations create regions of subtherapeutic exposure; hostile tumor conditions favor survival-oriented phenotypes; and cancer cells respond by reorganizing metabolic and stress-response pathways. These interacting pressures may therefore do more than permit resistant cells to remain within the tumor: they can actively select for adaptive states capable of surviving treatment and later contributing to tumor repopulation [4,5,7,21,25,30,32,33,38].

Within this framework, PGCCs and related stem-like populations represent specialized manifestations of a broader therapy-tolerant survival response. Their significance lies not simply in their resistance to treatment, but in their ability to integrate polyploidization, reduced proliferative dependence, metabolic flexibility, and stress tolerance within a single adaptive state. Understanding how these states emerge from multiscale tumor pressures is essential for identifying vulnerabilities that may be exploited through alternative therapeutic strategies [7,8,18,24,26,27].

The discussion presented here is intended to establish the multiscale context in which therapy-tolerant populations emerge rather than to provide an exhaustive review of tumor transport or microenvironmental biology. Readers seeking deeper analyses of drug penetration and transport within solid tumors are directed to references [32,35,36]. More comprehensive discussions of tumor microenvironmental heterogeneity, hypoxia, and metabolic adaptation can be found in references [4,21,30,33], while the quantitative limitations of nanoparticle delivery are examined in reference [36].

## 4. Limitations of Conventional Cancer Therapies in Therapy-Tolerant Cancer States

### 4.1. Conventional Therapies as Selective and Adaptive Stressors

As established in the earlier sections, substantial tumor reduction does not necessarily indicate eradication of therapy-tolerant residual populations. The discussion here therefore focuses more narrowly on how conventional therapies, including chemotherapy and radiation, impose mitotic, genotoxic, and oxidative stress that may eliminate the proliferating tumor bulk while selecting for or inducing adaptive cellular states [5,6,7,28,29]. These treatment-specific responses provide the context for examining how mitotic slippage, polyploidization, enhanced stress buffering, and other survival mechanisms can contribute to post-treatment persistence.

### 4.2. Therapy-Induced Stress Adaptation and Survival 

Several commonly used anticancer therapies illustrate these adaptive resistance mechanisms. Taxane-based therapies such as paclitaxel exert anticancer effects primarily through stabilization of microtubules and induction of prolonged mitotic arrest [41,42]. Although this process is intended to trigger mitotic catastrophe or apoptotic elimination, resistant cells may instead undergo mitotic slippage, allowing for escape from mitotic arrest and promoting formation of polyploid or multinucleated cell states associated with therapeutic persistence [43,44,45]. Experimental studies have demonstrated that cells escaping prolonged mitotic arrest may survive despite severe genomic instability, ultimately contributing to therapy resistance and post-treatment repopulation [42,46]. Importantly, mitotic slippage and therapy-induced polyploidization have been increasingly linked to formation of PGCC populations capable of surviving treatment exposure and later generating progeny through non-standard division mechanisms [7,18,24].

Similarly, platinum-based agents such as cisplatin induce extensive DNA damage through formation of DNA adducts, intra-strand crosslinks, inter-strand crosslinks, and replication stress [47,48,49]. While these effects can effectively eliminate highly proliferative tumor cells, surviving populations may persist through enhanced DNA repair activity, damage tolerance mechanisms, activation of stress-response pathways, and metabolic adaptation [47,50,51]. Several studies have further demonstrated that cisplatin-resistant populations frequently exhibit increased antioxidant capacity and altered glutathione metabolism, both of which contribute to stress tolerance and therapeutic persistence [51,52]. These residual populations may also enter dormant or quiescent states that reduce susceptibility to subsequent therapeutic exposure [7,23]. These dormant residual populations may later re-enter proliferative states following treatment cessation, thereby contributing to recurrence and long-term therapeutic failure [7].

Anthracyclines such as doxorubicin further illustrate the complex relationship between therapeutic stress and adaptive survival. Doxorubicin exerts anticancer activity through multiple mechanisms, including DNA intercalation, topoisomerase II inhibition, chromatin disruption, and generation of ROS [53,54]. While oxidative stress contributes significantly to cytotoxicity, surviving cancer cells may adapt through activation of antioxidant defenses, mitochondrial reprogramming, and stress-response signaling pathways that permit survival under continued therapeutic exposure [55,56,57]. Doxorubicin-induced ROS production has also been linked to broader metabolic and redox adaptations that may increase tolerance to oxidative injury over time [56,58]. These observations are particularly relevant because oxidative stress adaptation and redox buffering are increasingly recognized as central features of therapy-tolerant cancer states [18,38,59,60].

Radiation therapy similarly induces substantial oxidative and genotoxic stress through ROS generation and DNA damage [61,62]. Ionizing radiation can directly damage DNA while also indirectly producing ROS that contribute to oxidative injury, activation of DNA damage response pathways, and cell death [57,61]. However, resistant cancer cell populations may survive radiation exposure through activation of antioxidant buffering systems, DNA repair pathways, metabolic adaptation, and stress-response signaling capable of limiting lethal oxidative injury [57,61]. Importantly, several of these adaptive responses overlap significantly with biological features associated with therapy-tolerant populations, including persistence under hostile microenvironmental conditions, metabolic flexibility, and survival despite prolonged therapeutic exposure [7,18,39].

Additional chemotherapeutic classes further demonstrate that mechanistically distinct anticancer agents can converge on polyploidization or other therapy-tolerant survival states. Vinca alkaloids such as vincristine disrupt microtubule polymerization and spindle formation; resistance to anti-tubulin therapy can be accompanied by chemotherapy-induced polyploidy [63]. Antimetabolites such as 5-fluorouracil disrupt nucleotide metabolism and DNA synthesis, and experimental work in triple-negative breast cancer has linked 5-fluorouracil-based treatment to premature senescence and autophagic responses [64]. Etoposide, a topoisomerase II inhibitor, produces DNA double-strand damage and, at low doses, can induce a senescence-like phenotype accompanied by endoreplication and expansion of polyploid populations [65]. Although these agents initiate cytotoxicity through different mechanisms, their ability to generate non-proliferative or polyploid survivors supports the concept that therapy-tolerant states can emerge across multiple anticancer drug classes. These representative therapeutic and microenvironmental stressors and their associated adaptive responses are summarized in Table 2.

### 4.3. Therapy-Induced Selection Pressure and Residual Disease

Collectively, these observations suggest that therapeutic failure often reflects survival through adaptive reprogramming rather than resistance through a single static mechanism alone [5,6,7,25,29,38].

These limitations emphasize the need for therapeutic strategies capable of targeting adaptive survival dependencies that persist independently of continued proliferation or classical apoptotic sensitivity, particularly pathways associated with oxidative stress regulation, metabolic adaptation, and stress tolerance [30,68,69].

This framework helps explain why therapies that are highly effective against proliferating cells may still fail to prevent recurrence. Residual disease may not simply represent incomplete killing, but rather the survival of cells that have shifted into adaptive states less dependent on proliferation and classical apoptosis [6,7,18,24,25]. In this context, PGCCs are especially important because they embody several features of therapy tolerance simultaneously: cell-cycle disruption, polyploidization, stress survival, altered metabolism, and the potential for later tumor repopulation [7,18,24,25]. These observations support the need for therapeutic strategies that target the dependencies created by stress adaptation rather than relying exclusively on mechanisms designed to eliminate rapidly dividing cells [6,7,18,24,25,27,38]. 

## 5. Nanomedicine Strategies for Therapy-Tolerant Cancer States

### 5.1. Why Nanomedicine Is Relevant to Resistant Populations

Nanomedicine is relevant to these resistant populations because nanoparticle-based systems can be engineered to alter biodistribution, improve drug stability, increase tumor retention, enable combination delivery, and respond to tumor-associated stimuli such as acidic pH, hypoxia, elevated hydrogen peroxide, altered iron metabolism, or abnormal redox balance [70]. PGCCs represent stress-resilient cancer cell states whose adaptive survival characteristics may create opportunities for targeted therapeutic strategies [71]. Ferroptosis-oriented nanomaterials, including multifunctional nanoparticle platforms, offer potential approaches for exploiting tumor-associated redox and metabolic vulnerabilities [72,73,74,75]. However, this rationale must be framed carefully. Nanomedicine does not fully overcome tumor delivery barriers, and nanoparticle accumulation remains limited and heterogeneous across tumor types and models [36,74,76]. Rather than presenting nanoparticles as a complete solution to poor penetration, their translational value may lie in their ability to partially improve delivery while simultaneously exploiting biochemical vulnerabilities generated by therapy adaptation.

This distinction is particularly important for therapy-tolerant cancer states. Resistant cells are often difficult to eliminate because they may be slow-cycling, metabolically flexible, stress-adapted, and less dependent on pathways targeted by conventional cytotoxic therapies [25,38,59,60]. Yet, these same adaptive features may create liabilities, including increased dependence on antioxidant defenses, altered lipid metabolism, iron dysregulation, mitochondrial remodeling, and vulnerability to lipid peroxidation [38,59,60,77,78]. Therefore, the rationale for nanomedicine in this setting is not simply improved drug delivery; it is the possibility of designing platforms that connect delivery to the specific stress biology of therapy-tolerant populations.

**Figure 1:**
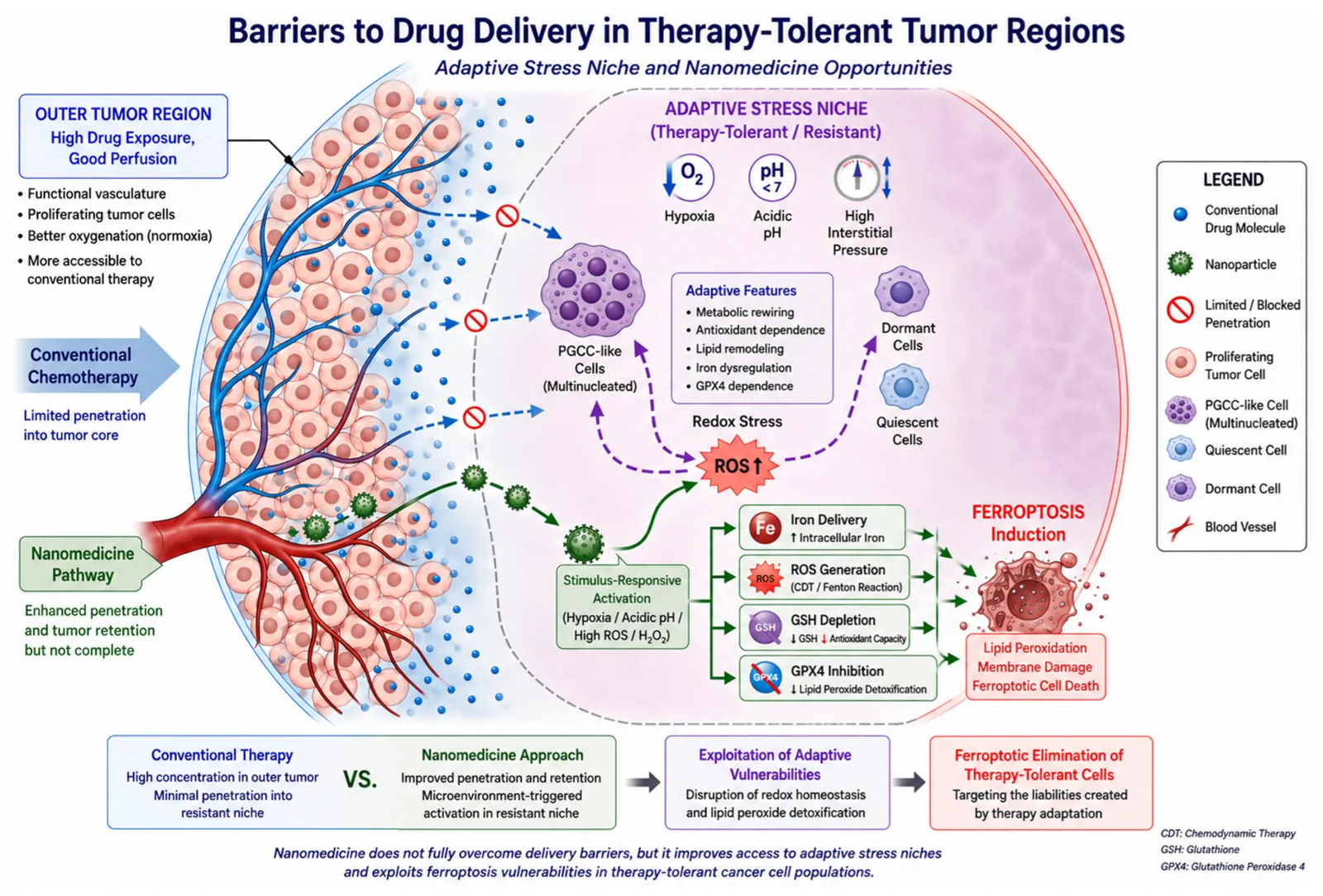
Barriers to drug delivery in therapy-tolerant tumor regions and potential nanomedicine opportunities. Blue arrows indicate conventional drug distribution, with dashed arrows and red inhibitory symbols representing restricted penetration into the tumor core. Green arrows indicate nanoparticle delivery, stimulus-responsive activation, and four potential ferroptosis-inducing mechanisms: iron delivery, ROS generation, GSH depletion, and GPX4 inhibition. Upward and downward arrows within the mechanism boxes indicate increased intracellular iron or decreased GSH availability, antioxidant capacity, and lipid peroxide detoxification, respectively. Purple dashed arrows illustrate conceptual relationships between redox stress and therapy-tolerant cellular states, while gray arrows represent progression through the proposed therapeutic framework. The four ferroptosis-inducing mechanisms represent potential strategies and do not necessarily occur simultaneously within a single nanoparticle formulation.

Conventional therapies often exhibit limited penetration into hypoxic and poorly perfused tumor regions where therapy-tolerant populations emerge and persist [32,33,34,35,36]. These adaptive stress niches are characterized by altered redox homeostasis, metabolic rewiring, antioxidant dependence, and elevated oxidative stress [38,59,60,77], features that may create exploitable vulnerabilities for ferroptosis-oriented nanomedicine approaches [70,72,73,74], as will be discussed in detail in the following sections. Consequently, increasing attention has been directed toward nanoparticle systems capable of inducing ferroptosis through iron delivery, ROS amplification, glutathione depletion, and GPX4 inhibition [21,25,32,33,34,36,38,59,60,70,72,73,74]. The relationship between restricted drug penetration, therapy-tolerant niches, and potential nanomedicine-enabled ferroptosis strategies is illustrated in Figure 1.

Building on the resistance and transport barriers described in Section 2 and Section 3, nanomedicine adds a delivery-based strategy by protecting unstable or poorly soluble agents, modifying pharmacokinetics and biodistribution, enabling alternative routes of cellular entry, and supporting co-delivery of therapeutics intended to overcome resistance mechanisms [9,10,11].

Nanomedicine may also help address resistant cancer stem-cell (CSC) populations, which are relevant to recurrence because they can remain quiescent or slow-cycling, overexpress drug-efflux transporters, and regenerate heterogeneous tumor cell populations after treatment [9,11,13]. Nanocarriers have been investigated as a means of delivering poorly soluble or otherwise pharmacologically constrained agents to CSCs, inhibiting efflux transporters, delivering siRNA or other nucleic-acid therapeutics, and targeting stem-associated surface markers such as CD44 or CD133 [9,11]. More recent work has extended this concept to metabolically adapted CSC states by designing pH-, hypoxia-, and metabolism-responsive platforms that exploit conditions enriched within stem-like tumor niches [13]. This literature is especially relevant to PGCCs because PGCCs can display stem-like and therapy-tolerant behavior; however, evidence established in CSC models should not be assumed to demonstrate PGCC-selective nanoparticle targeting.

As detailed in Section 3, the tumor microenvironment creates both biochemical and physical barriers to therapy. From a nanomedicine perspective, these features can be exploited through passive or ligand-directed targeting, prolonged circulation, and release triggered by low pH, hypoxia, redox gradients, reactive oxygen species, or tumor-associated enzymes; some platforms can also target tumor vasculature or stromal components rather than malignant cells alone [9,10,11,20].

The value of nanomedicine therefore depends less on the existence of a particular carrier class than on whether the formulation solves a defined resistance problem. Contemporary delivery strategies include conventional liposomes and polymeric systems, lipid nanoparticles, inorganic carriers, carrier-free assemblies, extracellular-vesicle or biomimetic platforms, and multifunctional systems that combine targeting, controlled release, and coordinated delivery of complementary agents [11,20]. At the same time, nanoparticle accumulation and penetration remain heterogeneous across tumors, and improved delivery in preclinical models does not guarantee mechanism-specific reversal of resistance in patients [20]. For therapy-tolerant PGCC-like states, the most relevant application is therefore to connect a necessary delivery function with a stress-associated vulnerability that free drugs cannot safely or effectively exploit. This principle provides the transition to ferroptosis-oriented nanomedicine, in which localization and coordinated manipulation of iron, reactive oxygen species, glutathione, and lipid-peroxide defenses may be as important as the cytotoxic payload itself. Representative nanoparticle platforms, therapeutic cargoes, and delivery approaches relevant to resistant cancer populations are summarized in Table 3.

### 5.2. Ferroptosis-Oriented Nanomedicine Platforms

**Figure 2:**
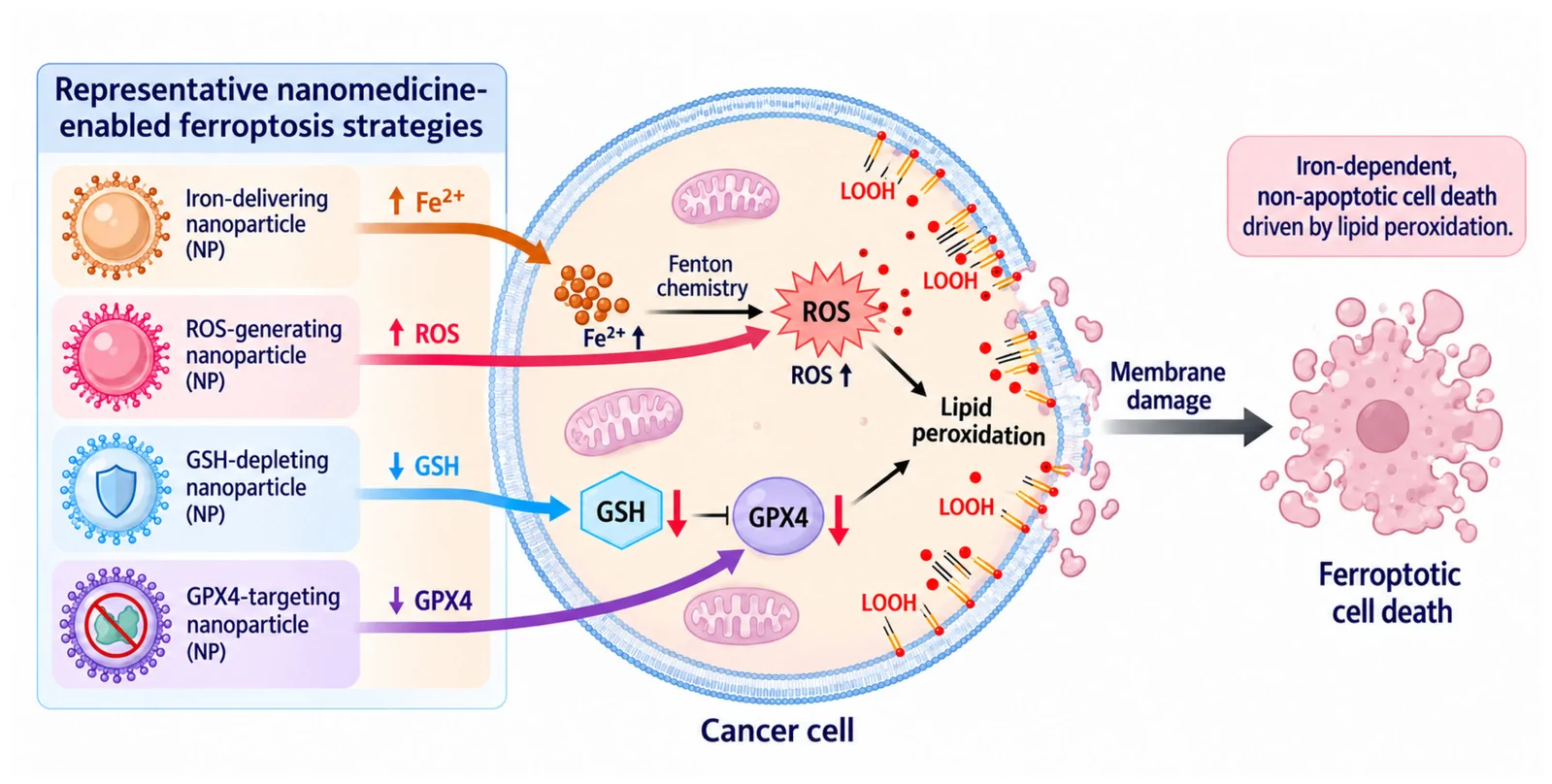
Cytotoxic mechanism of ferroptosis and representative nanomedicine-enabled strategies. Orange, pink, blue, and purple arrows indicate nanoparticle-mediated iron delivery, ROS generation, GSH depletion, and GPX4 inhibition, respectively. Upward and downward arrows indicate increased Fe²⁺ and ROS levels or decreased GSH and GPX4 activity. Black arrows illustrate the progression from iron-mediated Fenton chemistry and ROS generation to lipid peroxidation, membrane damage, and ferroptotic cell death. Red downward arrows indicate reduced GSH availability and GPX4 activity, impairing lipid peroxide detoxification. The depicted nanoparticle strategies represent potential mechanisms and do not necessarily occur simultaneously within a single formulation.

Ferroptosis is an iron-dependent, non-apoptotic form of regulated cell death driven by uncontrolled phospholipid peroxidation and failure of cellular antioxidant defenses, particularly the glutathione–GPX4 system [80,81,82]. This mechanistic distinction makes ferroptosis particularly relevant to therapy-tolerant cancer states, which may evade conventional treatment through altered apoptotic signaling, cell-cycle slowing, enhanced repair capacity, epithelial–mesenchymal transition (EMT), metabolic rewiring, and other adaptive survival programs [4,5,25,38]. Ferroptosis susceptibility is governed by the balance among iron availability, polyunsaturated phospholipid oxidation, reactive oxygen species generation, glutathione availability, and GPX4-mediated detoxification of lipid hydroperoxides [59,60,80,81]. Nanomedicine platforms may provide a means of therapeutically manipulating these processes within resistant tumor populations [70,72,73,80,81]. The core cytotoxic mechanism of ferroptosis and representative nanomedicine-enabled approaches that can amplify ferroptotic stress are illustrated in Figure 2.

A particularly important example comes from Hangauer et al. [59], who demonstrated that drug-tolerant persister (DTP) cancer cells develop a selective dependency on glutathione peroxidase 4 (GPX4), a central suppressor of ferroptosis. GPX4 inhibition preferentially eliminated these therapy-persistent cells through lipid peroxidation-dependent ferroptotic death, indicating that acquisition of drug tolerance can create a new metabolic vulnerability rather than simply producing broad resistance to cell death. This finding is significant because DTP cells are characterized by slow proliferation and reduced sensitivity to conventional cytotoxic mechanisms, features shared by several residual therapy-tolerant populations discussed in this review. Ferroptosis therefore provides a mechanistically distinct strategy for eliminating cells that survive initial treatment by shifting away from proliferation-dependent and apoptosis-dependent states. From a nanomedicine perspective, this dependency also provides a rationale for localized delivery of GPX4 inhibitors, glutathione-depleting agents, or complementary oxidative stress-inducing payloads to resistant tumor compartments where systemic exposure might otherwise limit therapeutic feasibility.

A complementary example was reported by Viswanathan et al. [60], who found that therapy-resistant cancer cells adopting a mesenchymal-like state exhibit increased dependence on lipid-peroxide detoxification and are highly susceptible to GPX4 inhibition. These findings are important because epithelial–mesenchymal plasticity is frequently associated with treatment resistance, invasion, stem-like behavior, and survival following therapeutic stress. Rather than eliminating resistance through restoration of sensitivity to the original therapy, ferroptosis exploits a liability created by the resistant state itself: increased dependence on mechanisms that prevent toxic lipid peroxidation. This concept is particularly relevant to heterogeneous tumors in which resistant cells may transition among stem-like, mesenchymal, dormant, and other adaptive phenotypes. Nanomedicine could enhance this strategy by concentrating ferroptosis-inducing agents within these resistant populations or by combining lipid-peroxide amplification with disruption of GPX4, glutathione, iron, or related antioxidant pathways in a single delivery system.

Additional mechanistic studies support this broader framework by linking DTP-cell survival to mitochondrial metabolism, PGRMC1-dependent lipophagy and fatty-acid oxidation, and FSP1-regulated iron and lipid homeostasis [37,77,78]. Collectively, these findings suggest that some therapy-tolerant cells persist through tightly maintained redox and lipid-defense programs that may become therapeutically exploitable when ferroptotic stress is selectively amplified.

A major category of ferroptosis-oriented nanomedicine involves iron-containing nanoparticles or iron-delivery platforms. These systems can increase intracellular iron availability and promote Fenton or Fenton-like reactions that convert endogenous hydrogen peroxide into highly reactive hydroxyl radicals, thereby increasing oxidative stress and lipid peroxidation [70,72,73,83,84]. Iron oxide nanoparticles, ferritin-based nanocarriers, metal-organic frameworks, and other iron-containing platforms have been investigated as tools to increase unbound iron pools and push tumor cells toward ferroptotic death [70,72,74]. These approaches are attractive because many tumor regions already contain elevated ROS, acidic pH, and altered iron metabolism, creating conditions that may support localized ROS amplification [21,33,70,72,73,74,84].

Chemodynamic therapy (CDT) is closely related to this concept. In CDT, transition metal-based nanomaterials catalyze ROS generation within the TME, often through Fenton or Fenton-like chemistry [70,72,73,84]. Because CDT relies on endogenous tumor features such as hydrogen peroxide and acidity, it is often framed as a tumor microenvironment-responsive approach rather than a therapy requiring uniform external activation. This is especially relevant to hypoxic or poorly perfused tumor regions, where conventional drug delivery and oxygen-dependent therapies may be limited [32,33,74]. By amplifying ROS within stress-adapted tumor regions, CDT-based nanomedicine may create oxidative pressure that exceeds the buffering capacity of resistant cells [38,59,60,70,72,73,74,84].

Another major strategy involves depletion of glutathione (GSH). Glutathione is a central intracellular antioxidant and an essential cofactor for GPX4, the enzyme responsible for reducing lipid hydroperoxides and suppressing ferroptosis [73,80]. Nanoparticles that consume GSH, disrupt GSH synthesis, deliver ferroptosis-inducing agents, or weaken antioxidant capacity can sensitize tumor cells to lipid peroxidation and ferroptotic death [70,72,73,74,84]. This mechanism is particularly relevant to therapy-tolerant cells because persister-like populations can depend heavily on GPX4 activity, redox buffering, and lipid peroxide repair for survival [37,59,60,77,78].

Combination nanomedicine platforms may be especially important because therapy-tolerant populations are biologically heterogeneous and are unlikely to be eliminated by a single mechanism. Multifunctional nanoparticles have been developed to combine ferroptosis induction with chemotherapy, photodynamic therapy, photothermal therapy, immunotherapy, radiotherapy, or metabolic therapy [70,72,73,74,75,84]. These platforms are conceptually attractive because they can coordinate multiple mechanisms within the same delivery system: increasing iron availability, generating ROS, weakening antioxidant defenses, and delivering cytotoxic or immune-modulating agents. In resistant tumors, this type of combination approach may be necessary because adaptive cell states can shift between quiescence, senescence-like survival, stem-like behavior, polyploidy, and proliferative re-entry [25,26,31,66,85].

Despite this promise, ferroptosis-oriented nanomedicine remains largely preclinical, and several translational barriers remain unresolved. These include limited nanoparticle accumulation in human tumors, heterogeneous intratumoral distribution, variable tumor redox states, potential off-target toxicity, differences between animal models and human tumors, and uncertainty regarding which resistant populations are most ferroptosis-sensitive in vivo [36,74,76,83]. Therefore, ferroptosis-oriented nanomedicine should be presented as a mechanistically promising strategy rather than an established solution. Its strongest rationale lies in the convergence between delivery engineering and the redox, lipid, and iron-related vulnerabilities of therapy-tolerant cancer states [36,38,59,60,70,72,73,74,76,77,78,83].

### 5.3. Translational Rationale for PGCC-like States

Although most ferroptosis-oriented nanomedicine platforms were not developed specifically to target PGCCs, their mechanisms intersect with adaptive survival features associated with PGCC-like states. Evidence that PGCCs can persist following severe therapeutic and microenvironmental stress and subsequently contribute to tumor repopulation provides a rationale for investigating strategies that target stress-dependent survival rather than proliferation alone [7,18,24,26,27,31,39,66,67,71]. Ferroptosis-oriented nanomedicine is particularly relevant in this context because it may combine controlled delivery with disruption of the redox and lipid-homeostatic processes that support therapy-tolerant states.

Because PGCC-like cells may arise or persist within hypoxic, poorly perfused, acidic, and oxidatively stressed tumor regions where conventional therapy is least effective, partial delivery remains an important component of this translational rationale [31,32,33,71]. The distinction between whole-tumor nanoparticle accumulation and delivery to PGCC-containing niches is addressed in Section 6.4.

Importantly, PGCC-like cells should not be viewed as resistant simply because they are inherently “harder to kill.” Instead, their ability to survive hostile conditions may create biological tradeoffs. Cells that persist under hypoxic, oxidative, genotoxic, and nutrient-deprived conditions may become increasingly dependent on redox regulation, lipid remodeling, mitochondrial adaptation, iron handling, and antioxidant defense pathways [30,38,59,60,77,78]. Ferroptosis-oriented nanomedicine platforms may therefore be relevant because they can disrupt these systems through iron delivery, reactive oxygen species amplification, glutathione depletion, and GPX4 inhibition [70,72,73,74,84].

At present, however, direct evidence that ferroptosis-inducing nanoparticles selectively eliminate PGCCs remains limited [18,70,72,73,74,75,86,87]. Accordingly, the argument is not that ferroptosis-oriented nanomedicine is already a validated PGCC-targeting strategy. Rather, PGCC-like states appear to share adaptive features with broader therapy-tolerant populations, including redox dependence, lipid peroxide defense, altered iron handling, autophagy, and metabolic stress tolerance, that future nanomedicine platforms could potentially exploit [7,18,38,39,59,60,67,71,72,73,74,77,78].

Taken together, ferroptosis-oriented nanomedicine provides a conceptual bridge between delivery engineering and the stress-adapted biology of therapy-tolerant cancer cells. For PGCC-like states specifically, the priority is to move beyond general associations with therapy resistance and directly determine whether PGCC-enriched populations exhibit altered iron metabolism, GPX4 dependence, glutathione reliance, lipid peroxide sensitivity, or differential responses to ferroptosis-inducing nanomedicine platforms.

## 6. Polyploid Giant Cancer Cells as Therapy-Tolerant States: Vulnerabilities, Ferroptosis, and Nanomedicine

### 6.1. PGCCs as Stress-Adapted Therapy-Tolerant States

Building on the broader framework of therapy tolerance introduced in the prior sections, PGCCs merit focused consideration because their persistence following therapeutic stress and their capacity for noncanonical progeny generation may connect residual disease with subsequent tumor repopulation [18,24]. PGCCs have also been identified in patient tumor samples, including after chemotherapy and radiation exposure, supporting their relevance to post-treatment residual disease beyond experimental systems [7,18]. Although much of the mechanistic evidence remains preclinical, their presence within treated tumors indicates that PGCCs may represent a clinically relevant component of therapy-tolerant disease [7,18,26,66,71,88,89].

PGCCs exhibit molecular and functional characteristics that distinguish them from normally proliferating tumor cells. These include dysregulation of cell-cycle checkpoints, activation of DNA damage-response pathways, enhanced autophagic activity, metabolic reprogramming, and increased expression of stemness-associated markers [7,18,24,39,67]. Alterations involving p53, PI3K/Akt/mTOR, HIF-1α, and Wnt/β-catenin signaling have also been implicated in adaptation to therapeutic stress and long-term persistence [7,18,24,67]. In addition, PGCCs have been associated with increased oxidative-stress tolerance, altered lipid metabolism, and enhanced survival signaling [18,39]. These features suggest that PGCC persistence reflects adaptive reprogramming rather than classical resistance alone and may therefore depend on biological systems that create therapeutically exploitable vulnerabilities.

This distinction is important because the PGCC state is not necessarily a terminal endpoint. Following removal of therapeutic stress, surviving PGCCs can generate proliferative progeny through noncanonical division, linking transient growth arrest with later tumor repopulation [7,18,24]. Their therapeutic significance therefore lies not only in their ability to remain viable during treatment, but also in their potential to preserve malignant capacity until conditions permit renewed growth. PGCCs can consequently be viewed as stress-adapted cellular reservoirs within residual disease rather than passive remnants of treatment-induced injury [7,18,24,26,27,31]. 

Recent mechanobiological evidence further supports the interpretation of PGCCs as active therapy-tolerant states rather than terminally damaged cells. Ku et al. [19] demonstrated that radiation-induced PGCCs in triple-negative breast cancer develop a distinct state of “mechanical dormancy,” characterized by cellular and nuclear enlargement, cortical actin remodeling, increased mechanical stiffness, and suppressed proliferation. Importantly, invasion-associated molecular features remained sustained despite reduced proliferative activity, suggesting that dormancy does not necessarily represent biological inactivity. Similar PGCC-associated morphological and mechanical changes were observed in irradiated xenograft tumors, providing in vivo support for persistence of this phenotype following therapeutic stress. These findings broaden the concept of PGCC adaptation beyond metabolic and redox reprogramming by showing that therapy tolerance can also involve coordinated changes in cellular mechanics and cytoskeletal organization [19].

Accordingly, the central question is not simply whether PGCCs survive treatment, but which adaptive mechanisms make their persistence possible and whether those mechanisms can be disrupted. Framing PGCCs in this way preserves their role as a specialized therapy-tolerant state while directing attention toward the dependencies that distinguish survival under stress from irreversible treatment-induced damage [7,18,24,27,39,67].

These endpoints should be interpreted separately. A reduction in the number of morphologically identifiable PGCCs may reflect inhibition of PGCC formation, loss of established PGCC viability, or both, but does not by itself demonstrate prevention of tumor repopulation. Stronger evidence of functional PGCC targeting requires assessment of whether surviving PGCCs subsequently generate proliferative progeny and, ultimately, whether treatment reduces long-term regrowth or recurrence. Accordingly, the studies discussed below are distinguished according to whether they evaluate PGCC formation, PGCC survival, daughter-cell production, or tumor-level regrowth.

### 6.2. Vulnerabilities Within PGCC Survival Programs

The ability of PGCCs to withstand therapeutic stress does not necessarily make them invulnerable. Their persistence under conditions of chronic metabolic, genotoxic, and oxidative stress may require a tightly maintained adaptive equilibrium involving redox regulation, membrane integrity, organelle quality control, and nutrient recycling [7,18]. The same programs that support PGCC survival may therefore create dependencies that can be therapeutically targeted.

One potential vulnerability involves redox homeostasis. Chemotherapy, radiation, hypoxia, and nutrient deprivation can generate persistent oxidative pressure [18,39]. To remain viable under these conditions, PGCCs may depend on antioxidant defenses, stress-response signaling, and metabolic adaptations that prevent the accumulation of lethal oxidative damage. Although these systems promote survival, they may also increase dependence on mechanisms that maintain intracellular redox balance. Disrupting oxidative-stress buffering may therefore destabilize therapy-tolerant PGCC populations without requiring renewed proliferative activity [18,38,39,71].

Alterations in lipid metabolism and membrane remodeling may represent a second vulnerability. Therapy-tolerant cancer states frequently exhibit changes in lipid synthesis, membrane composition, and lipid utilization that support survival during prolonged stress [18,39]. In PGCCs, these adaptations may help maintain enlarged cellular and organelle architecture but could also increase dependence on lipid-peroxide detoxification. This relationship is particularly relevant to ferroptosis, which is driven by the accumulation of oxidized polyunsaturated phospholipids within cellular membranes. Lipid remodeling that supports PGCC persistence may therefore simultaneously create susceptibility to strategies that promote lipid peroxidation or impair its detoxification [18,38,39,59,60,77,78,80,82,90].

Autophagy may provide an additional survival dependency [39,67]. By recycling damaged proteins, organelles, and other cellular components, autophagy supports energy homeostasis and limits the accumulation of intracellular damage during nutrient deprivation, hypoxia, chemotherapy exposure, and oxidative stress [39,40,67]. You et al. demonstrated that AMPK-mTOR-mediated autophagy promotes formation of dormant PGCC populations, while Patra et al. identified autophagy as an important contributor to PGCC persistence, stress tolerance, and metabolic adaptation [39,67]. These findings support a role for autophagy in both PGCC formation and continued survival by maintaining metabolic flexibility and cellular quality control under prolonged therapeutic stress.

These adaptive processes are unlikely to function independently. Redox regulation, lipid metabolism, mitochondrial function, stress-response signaling, and autophagy form an interconnected survival network. Their convergence on oxidative-stress regulation, lipid-peroxide control, and metabolic homeostasis provides the mechanistic basis for investigating ferroptosis as a strategy for targeting therapy-tolerant PGCC populations [7,18,38,39,59,60,67,68,69,77,78,90].

### 6.3. Ferroptosis as a Mechanistically Aligned Therapeutic Strategy

The potential relevance of ferroptosis to therapy-tolerant cancer populations stems from its mechanistic distinction from conventional apoptosis-dependent approaches. Whereas many standard anticancer therapies rely on proliferative vulnerability and activation of apoptotic pathways, ferroptosis is a non-apoptotic form of regulated cell death driven by iron-dependent accumulation of membrane lipid peroxides and failure of antioxidant defenses [69,80,81,82]. It may therefore remain actionable in cellular states characterized by reduced proliferative activity or impaired classical apoptotic signaling.

Ferroptosis directly exploits antioxidant dependence by overwhelming lipid-peroxide defense. GPX4 reduces lipid hydroperoxides and prevents propagation of oxidative membrane damage; inhibition of GPX4 activity or depletion of intracellular glutathione can permit uncontrolled lipid peroxidation and ferroptotic death [80,82]. This relationship is particularly relevant to therapy-tolerant populations because drug-tolerant persister and mesenchymal-like resistant states have demonstrated dependence on GPX4-mediated lipid-peroxide detoxification [59,60]. Such findings do not establish identical behavior in every PGCC population, but they provide a mechanistic precedent for targeting stress-adapted cells through the antioxidant systems required for their persistence.

PGCC-associated lipid remodeling and autophagic dependence provide additional points of convergence. Membrane lipid composition governs susceptibility to phospholipid peroxidation [80,81], while autophagy supports PGCC formation and persistence [39,67]. Selective autophagic processes such as ferritinophagy can also increase the pool of redox-active iron available to drive lipid peroxidation [82,91,92]. Therefore, pathways that preserve PGCC viability under prolonged stress may also determine the threshold at which ferroptotic injury becomes irreversible.

Ferroptosis is also attractive in this context because it does not depend on active proliferation. Conventional cytotoxic agents often exhibit reduced efficacy against dormant, quiescent, or slowly proliferating cancer cells because their mechanisms depend heavily on cell division and DNA replication [7,23]. By contrast, ferroptosis targets metabolic and redox functions that may remain essential regardless of proliferative status. Ferroptosis-oriented approaches could therefore complement conventional treatment by targeting residual populations after the proliferating tumor bulk has been reduced, rather than serving as a direct replacement for existing modalities [25,59,60,69,80,81,82].

Importantly, ferroptosis is not the only strategy under investigation for disrupting PGCC-mediated persistence. Zhang et al. showed that sublethal olaparib exposure induced PGCC formation in ovarian and breast cancer cell lines, high-grade serous ovarian cancer-derived organoids, and patient-derived xenografts. After olaparib withdrawal, PGCCs generated mitotically competent, olaparib-resistant daughter cells through noncanonical division. Mifepristone inhibited PGCC formation and daughter-cell production; in patient-derived xenograft models, mifepristone combined with olaparib reduced tumor growth, while mifepristone alone suppressed tumors with acquired olaparib resistance [93]. Although this mechanism is distinct from ferroptosis, the study provides direct proof-of-concept that interfering with PGCC formation or reproductive recovery can improve therapeutic response and supports PGCCs as actionable rather than merely descriptive treatment-associated phenotypes.

The relevance of ferroptosis to PGCC-like states is therefore not simply that ferroptosis is non-apoptotic. Rather, ferroptosis targets the same biological systems that therapy-tolerant cells may rely on for survival under stress: antioxidant buffering, glutathione availability, GPX4 activity, iron handling, mitochondrial metabolism, and lipid-peroxide detoxification. Current evidence supports this mechanistic convergence, although the extent to which ferroptosis sensitivity is conserved across PGCC populations remains incompletely defined. Much of the broader rationale is informed by therapy-resistant, mesenchymal, stem-like, and metabolically adapted cancer states [59,60,90], emphasizing the importance of continued PGCC-specific validation [37,38,59,60,77,78,80,82,90]. The relative strength of the evidence discussed in these sections, including whether PGCCs were directly studied, is summarized in Table 4.

### 6.4. Delivery Considerations for Exploiting PGCC-Associated Vulnerabilities 

Mechanistic alignment alone is insufficient if therapeutics cannot reach residual populations at effective concentrations. PGCCs and related therapy-tolerant states may persist within poorly perfused, spatially heterogeneous tumor regions characterized by hypoxia, elevated interstitial pressure, abnormal vasculature, and restricted drug penetration [32,35,36]. These conditions reduce therapeutic exposure while simultaneously supporting the stress-adapted states that survive treatment [7,18]. Nanomedicine is therefore relevant not as a complete solution to tumor transport barriers, but as a potential means of linking controlled delivery with the biochemical vulnerabilities created by therapy adaptation [70,72,73,74,96,97].

Delivery is especially important for ferroptosis-oriented therapy because the intended mechanisms; iron dysregulation, ROS amplification, glutathione depletion, and GPX4 inhibition, can also injure normal tissues when exposure is not adequately localized [16,73,74,98]. Small-molecule ferroptosis inducers may additionally face limited solubility, rapid clearance, insufficient intracellular accumulation, or a narrow therapeutic window [73,74,79,96,97]. A delivery platform that controls where, when, and in what combination these stresses are produced may therefore be as important as the identity of the ferroptosis-inducing payload itself [16,17,73,74,79,83,96,97,98].

Several biological features associated with PGCCs may provide opportunities for nanoparticle-based targeting. Reported features include altered membrane composition, elevated oxidative stress, enhanced endocytic or nutrient-scavenging behavior, and dependence on autophagy and metabolic adaptation [18,39]. Stimulus-responsive nanoparticles could be designed to release payloads in response to acidic pH, elevated ROS, hypoxia, or other conditions enriched within stress-adapted tumor niches [70,72,73,84,96,97]. Molecular characteristics associated with PGCCs, including stemness-associated markers and altered metabolic signaling, may eventually support more selective targeting; however, direct PGCC-specific targeting strategies remain largely unexplored [7,18,26,39,66,71,72,73,74,84,88,96,97,99].

The rationale for nanomedicine must remain appropriately qualified. Nanocarriers do not uniformly overcome poor tumor penetration and continue to face heterogeneous accumulation, immune clearance, variable intratumoral distribution, off-target exposure, and long-term safety limitations [36,79]. Their principal value in this setting may lie in improving localization and controlled exposure sufficiently to enable ferroptosis-oriented strategies that would otherwise produce inadequate tumor concentrations or unacceptable systemic oxidative toxicity [79,96,97].

Importantly, increased total tumor accumulation does not establish effective delivery to PGCC-containing niches. Measurements based on whole-tumor drug or nanoparticle concentration may obscure substantial spatial heterogeneity in intratumoral distribution. Future PGCC-directed nanomedicine studies should therefore quantify penetration relative to vascular distance, hypoxic or acidic regions, and spatially identified PGCC populations rather than relying solely on bulk tumor accumulation. Stimulus-responsive release triggered by acidic pH, hypoxia, elevated ROS, or other niche-associated conditions may improve local activation after nanoparticles reach these regions, but such designs do not eliminate the underlying transport barrier itself [32,35,36,70,72,73,74,79].

For example, Naguib et al. evaluated PEGylated polymeric nanoparticles containing the ciprofloxacin derivative CIP2b in combination with paclitaxel in a treatment-resistant type II endometrial cancer model. The nanoparticle formulation increased intratumoral drug accumulation and prolonged systemic circulation, while combined treatment suppressed tumor progression without apparent off-target toxicity in the evaluated mouse model [15]. Although this system was not designed specifically for PGCCs or ferroptosis, it demonstrates how nanocarrier engineering can improve exposure to an otherwise pharmacologically constrained payload. More broadly, nanoparticle platforms can co-deliver iron donors, ferroptosis inducers, ROS-generating components, glutathione-depleting agents, or complementary stress-modulating payloads, enabling coordinated disruption of multiple survival pathways [73,79,96,97].

### 6.5. Nanotechnology Strategies Potentially Relevant to PGCC-like States

Nanotechnology strategies potentially relevant to PGCC-like states are most usefully organized by therapeutic function rather than by individual platform. Iron-containing nanoparticles and nanocarriers that deliver ferroptosis inducers can increase intracellular iron, enhance Fenton chemistry, inhibit GPX4, or weaken glutathione-dependent defenses, thereby promoting lipid-peroxide accumulation [69,73,96,97]. These functions are mechanistically relevant because PGCC persistence has been associated with altered redox balance, lipid metabolism, autophagy, and other stress-buffering pathways [14,18,39,71], while related therapy-tolerant states show dependencies involving glutathione, GPX4, iron handling, and lipid-peroxide defense [37,38,59,60,77,78].

A second functional category involves localized ROS amplification. Photodynamic, sonodynamic, and chemodynamic systems can generate oxidative stress within the tumor microenvironment, while iron-containing chemodynamic platforms may simultaneously act as ROS-generating catalysts and ferroptosis inducers [68,73,96,97,100]. The significance of these systems is not simply their capacity to generate ROS, but their potential to impose oxidative pressure within tumor regions where therapy-tolerant cells are already dependent on narrowly maintained antioxidant defenses. Platforms that combine catalytic ROS production with glutathione consumption are particularly attractive because they increase oxidative burden while simultaneously weakening the systems responsible for neutralizing it [38,59,60,70,72,73,74,77,78,84,96,97,100].

Additional platforms may weaken compensatory metabolism or cellular recycling. Experimental examples include inhibition of glutathione synthesis with agents such as buthionine sulfoximine, disruption of mitochondrial or fatty-acid metabolism, and delivery of autophagy-modulating payloads alongside ferroptosis sensitizers [73,96,97]. In this framework, combinations should unite complementary stress-disrupting functions, such as an iron source with a glutathione-depleting component or a ferroptosis inducer with an autophagy-modulating payload, rather than simply adding another proliferation-dependent cytotoxic agent. Autophagy-directed systems are particularly relevant because autophagy contributes to PGCC formation and persistence, although the effect of autophagy modulation on ferroptosis may depend on cellular context [39,67].

Direct evidence demonstrating selective efficacy of these nanotechnology approaches against PGCCs remains limited [99]. Most systems have been evaluated against bulk tumors or generalized therapy resistance rather than isolated or enriched PGCC populations [18,39,71,88]. As a result, these platforms should be viewed as candidates for PGCC-specific testing. The critical experiments are not only whether a nanoplatform reduces total tumor volume, but whether it reaches PGCC-enriched niches, disrupts the intended redox or metabolic dependency, prevents daughter-cell generation, and reduces recurrence-producing residual disease [18,71,74,88,99].

### 6.6. Critical Synthesis: Ferroptosis-Oriented Nanomedicine as a Potential Strategy for Therapy-Tolerant PGCC-like States

Viewed collectively, the therapeutically relevant feature of PGCC biology may not be dependence on any single survival pathway, but dependence on the stability of an adaptive equilibrium. Antioxidant defenses, iron regulation, lipid remodeling, autophagy, mitochondrial adaptation, and organelle quality control collectively allow PGCCs to tolerate simultaneous genomic, oxidative, metabolic, and structural stress [7,18,39]. Reliance on these compensatory systems may narrow the range over which cellular homeostasis can be maintained. Excessive lipid peroxidation, depletion of antioxidant capacity, or disruption of iron-dependent redox control could therefore destabilize the therapy-tolerant state itself rather than merely inhibit one conventional resistance mechanism [68,69,90].

This framework does not require the assumption that all PGCCs are uniformly ferroptosis-sensitive. PGCC populations are likely heterogeneous, and much of the current rationale is informed by broader drug-tolerant persister, mesenchymal, stem-like, and metabolically adapted states [59,60,90]. The strength of the hypothesis lies in a testable mechanistic proposition: PGCC survival under sustained stress may create measurable dependencies on GPX4, glutathione, iron handling, lipid-peroxide detoxification, autophagy, or related buffering systems, and those dependencies may be selectively disturbed [7,18,39,59,60,66,67,71,77,78,90].

Ferroptosis susceptibility is therefore unlikely to be uniform across PGCC populations. Differences in phospholipid composition and the availability of peroxidizable polyunsaturated fatty acids can influence the extent of lipid-peroxide accumulation, while variation in iron uptake, storage, ferritin turnover, and export may alter the labile iron pool available to support ferroptotic reactions [80,81,82,91,92]. PGCC populations may also differ in glutathione availability, GPX4 activity, FSP1-associated defense, and other compensatory antioxidant systems capable of limiting lipid-peroxide propagation [59,60,78]. Consequently, the same stress-adapted features that create ferroptosis vulnerability in one PGCC population may confer relative resistance in another. Identifying these differences will be necessary to define predictive biomarkers of PGCC ferroptosis sensitivity.

Rather than viewing ferroptosis simply as an alternative cell-death pathway, the available literature supports a broader model in which ferroptosis may function as a metabolic failure mechanism capable of destabilizing the adaptive equilibrium that permits survival after treatment [38,59,60,73,77,78,80,81,82]. Nanomedicine may provide an enabling platform for localizing and combining the stresses needed to induce that failure within resistant tumor regions [70,72,73,75,79,96,97]. The resulting strategy is therefore not proliferation-centered: it seeks to exploit the biological cost of remaining viable under therapeutic stress [37,38,59,60,77,78].

### 6.7. Translational Proof-of-Concept: Zoledronic Acid Nanoparticles

A translational proof-of-concept is provided by studies investigating zoledronic acid (ZA) as a strategy for targeting therapy-tolerant cancer populations. Adibi et al. demonstrated that ZA reduced cisplatin-induced PGCC populations and altered metabolic characteristics associated with PGCC survival, including lipid accumulation, mitochondrial density, and ROS regulation [14]. These findings are important because they show that a clinically available agent can affect PGCC-enriched populations and support the broader premise that PGCC persistence can be disrupted through interference with stress-adaptive metabolism.

Clinical application of ZA against extraskeletal therapy-tolerant tumors is limited by its strong affinity for hydroxyapatite and rapid sequestration within bone [95]. Li and colleagues initially developed a bisphosphonate-metal complex-based nanoparticle formulation that altered ZA biodistribution, prolonged circulation, and reduced preferential bone accumulation relative to conventional administration [94]. They subsequently engineered ZA-containing nanoparticles with minimized premature drug release, resulting in greater extraskeletal tumor accumulation and improved antitumor activity relative to free ZA [95]. By maintaining drug encapsulation during circulation and improving delivery to tumor tissue, these studies demonstrate how rational nanoparticle engineering can address a pharmacokinetic limitation that would otherwise restrict a potentially useful PGCC-directed agent [14,94,95].

Importantly, these studies address separate components of the proposed strategy and should not be interpreted as evidence that ZA nanoparticles selectively eliminate PGCCs. Adibi et al. evaluated the effects of ZA on chemotherapy-induced PGCC populations, whereas the ZA nanoparticle studies by Li et al. focused on pharmacokinetics, biodistribution, extraskeletal tumor accumulation, and antitumor activity rather than PGCC-specific efficacy [14,94,95]. Furthermore, ferroptosis was not established as the mechanism of ZA-mediated cytotoxicity in these studies. The ZA example therefore serves as a proof-of-concept for overcoming a delivery limitation associated with a PGCC-relevant agent, not as direct evidence of ferroptosis-oriented PGCC nanotherapy.

Taken together, the ZA studies connect the two central requirements developed throughout this section: identification of a vulnerability that affects PGCC-associated phenotypes and development of a delivery strategy capable of producing therapeutically meaningful exposure outside bone [14,94,95]. They do not establish ZA nanoparticles as a validated PGCC therapy, but they provide a concrete example of how nanomedicine can convert a biologically relevant yet pharmacokinetically constrained agent into a more plausible strategy for targeting therapy-tolerant disease.

## 7. Challenges and Future Directions

Despite increasing recognition that therapy-tolerant cancer cell states contribute to treatment failure and recurrence, several major challenges remain before these concepts can be translated into clinically effective therapeutic strategies. A central limitation is the lack of standardized methods for identifying, isolating, and studying therapy-tolerant populations such as PGCCs across tumor types [7,66,71,88,99]. A major challenge in translating PGCC-directed strategies is that PGCCs remain biologically heterogeneous and are often identified by morphology, ploidy, or stress-adapted behavior rather than by a single validated molecular marker [7,66,71,88,99]. This lack of standardized identification makes it difficult to compare studies directly and limits the ability to determine whether PGCCs represent a distinct therapeutic target or one part of a broader continuum of stress-adapted cancer cell states.

A second major challenge is that direct evidence connecting PGCCs to ferroptosis sensitivity, although recently emerging, remains limited to a small number of preclinical studies [86,87]. The mechanistic rationale remains strong because PGCCs and related therapy-tolerant states exhibit alterations in oxidative stress regulation, metabolism, and lipid homeostasis that may create ferroptosis-relevant vulnerabilities [7,14,18,39,71]. Zhou et al. used high-throughput single-cell morphological screening and transcriptomic analysis in breast cancer models to identify ferroptosis inducers as inhibitors of PGCC survival [86]. In particular, RSL3 and ML162 reduced PGCC populations generated by taxane or Aurora kinase inhibitor treatment across multiple breast cancer cell lines [86]. The investigators also found elevated reactive oxygen species in PGCCs, while experimental modulation of ROS altered their response to RSL3, supporting oxidative stress as a determinant of ferroptosis sensitivity [86]. In a subsequent study presented in 2024, Zhou et al. reported that PGCCs accumulated following irradiation and that treatment with several ferroptosis inducers—including imidazole ketone erastin, RSL3, FINO2, ML162, and ML210—reduced irradiation-induced PGCC populations [87]. Together, these studies provide direct proof-of-concept that PGCCs can exhibit ferroptosis susceptibility. Further studies should determine whether this vulnerability is conserved across tumor types, establish its molecular determinants, validate selective PGCC eradication in vivo, and assess whether ferroptosis-oriented nanomedicine can effectively reach and eliminate PGCC-enriched populations [86,87]. These findings demonstrate reductions in treatment-induced PGCC populations, but they do not by themselves establish prevention of PGCC-derived daughter-cell production, long-term tumor regrowth, or recurrence.

Delivery remains another important translation barrier. Therapy-tolerant populations often persist in poorly perfused, hypoxic, acidic, and metabolically stressed tumor regions where conventional drug penetration is limited [32,34,35]. Nanomedicine may partially address these challenges by improving drug localization, protecting therapeutic payloads, enabling stimulus-responsive release, and allowing for combination delivery [70,96,97,101]. Despite the promise of nanoparticle-based delivery, clinical translation remains limited by tumor heterogeneity, poor penetration into poorly perfused tumor regions, biodistribution concerns, and the relatively low fraction of administered nanoparticles that reach solid tumors [32,35,36,70,76,101]. However, nanoparticle accumulation remains heterogeneous across tumor types and models, and systemic delivery is still limited by immune clearance, renal or hepatic elimination, variable tumor penetration, and off-target distribution [36,76,97]. Therefore, future nanomedicine studies should evaluate not only whether ferroptosis-inducing platforms reduce bulk tumor growth, but also whether they reach and eliminate residual therapy-tolerant populations within protected tumor niches.

Safety is also a major concern. Ferroptosis-oriented strategies depend on amplifying oxidative stress, increasing iron-dependent lipid peroxidation, or disrupting antioxidant defenses [69,98]. While these mechanisms may be useful for targeting resistant tumor cells, they also raise concerns regarding injury to normal tissues that are sensitive to oxidative damage [17,69,98]. This is especially important for nanoparticles capable of inducing ferroptosis through iron dysregulation, ROS generation, glutathione depletion, or inflammatory signaling [16,70,97]. Future platforms will need to balance therapeutic efficacy with tumor selectivity, carefully assessing biodistribution, long-term toxicity, immune activation, and potential effects on normal tissues [16,17,73,74,79,83,98].

Another important future direction is biomarker development. If ferroptosis-oriented nanomedicine is to be used against therapy-tolerant residual disease, biomarkers will be needed to identify patients and tumors most likely to benefit. Potential biomarkers, derived from the mechanisms reviewed here, may include markers of PGCC enrichment, oxidative stress, lipid metabolism, iron handling, GPX4 dependence, glutathione metabolism, autophagy activation, or therapy-induced dormancy [7,59,69,70]. These markers could help determine when ferroptosis-oriented strategies should be used, whether as part of initial combination therapy or as an adjuvant approach following conventional treatment.

Emerging biophysical approaches may complement molecular biomarkers for identifying therapy-tolerant residual disease. Ku et al. [19] identified reproducible changes in cortical stiffness and cytoskeletal organization in radiation-induced PGCCs, suggesting that nanomechanical single-cell profiling may provide an additional framework for recognizing or stratifying mechanically dormant, therapy-resistant populations in future studies. Because this approach remains preclinical, validation in patient-derived specimens and more complex translational models will be necessary before mechanical phenotypes can be used for treatment selection.

Establishing PGCC-selective efficacy will require experimental designs that move beyond short-term cytotoxicity. PGCCs generated from a defined parental population should be compared directly with matched non-PGCC tumor cells to determine whether ferroptosis sensitivity and nanoparticle uptake are selectively increased. Nanoparticle studies should quantify cellular internalization, intracellular payload release, and spatial delivery to PGCC-containing tumor regions. Ferroptotic death should be validated using established mechanistic markers and rescue with ferroptosis inhibitors rather than inferred from viability loss alone. Finally, long-term washout and regrowth assays should determine whether treated PGCCs retain the capacity to generate proliferative daughter cells, and in vivo studies should assess whether PGCC-directed treatment reduces subsequent tumor repopulation or recurrence.

Finally, the clinical positioning of these strategies must be considered carefully. Ferroptosis-oriented nanomedicine is unlikely to function as a single universal solution for therapy resistance. Instead, its most realistic role may be as part of a combination or sequential treatment strategy designed to eliminate residual adaptive populations that survive chemotherapy, radiation, or targeted therapy [69,70,96,97]. In this framework, conventional therapies may reduce proliferating tumor burden, while ferroptosis-oriented or stress-disrupting nanomedicine platforms may target the metabolic and redox dependencies of therapy-tolerant cells that remain after initial treatment. This approach aligns with the broader goal of preventing recurrence by addressing residual disease rather than focusing exclusively on short-term tumor shrinkage [25,38,59,60,69,70,72,73,74,75,96,97]. 

## 8. Conclusions

Therapy resistance cannot be fully explained by genetic resistance or incomplete cytotoxicity alone. Increasing evidence suggests that cancer cells can survive treatment by entering adaptive, therapy-tolerant states characterized by dormancy, quiescence, stem-like behavior, metabolic flexibility, and stress-response activation [5,7,22,23,25]. Within this framework, PGCCs represent a particularly important example of a stress-adapted cellular state because they challenge the assumption that enlarged, polyploid, or non-proliferating cells are biologically irrelevant. Rather than serving only as markers of treatment-induced damage, PGCCs may function as persistent reservoirs capable of surviving therapeutic stress and contributing to later tumor repopulation [7,71,88,89]. 

The central argument of this review is that the same adaptive programs that allow PGCCs and related therapy-tolerant populations to survive may also create therapeutic vulnerabilities. Redox buffering, lipid remodeling, iron regulation, autophagy, mitochondrial adaptation, and antioxidant dependence all support survival under hostile conditions, but they may also make these cells vulnerable to strategies that disrupt metabolic and oxidative equilibrium [59,60,69,70]. Ferroptosis is particularly relevant because it bypasses classical apoptosis and targets iron-dependent lipid peroxide accumulation, a process closely linked to the stress-adapted biology of therapy-tolerant cancer states [69,70,81,97]. 

Nanomedicine provides a potential platform for exploiting these vulnerabilities, not because nanoparticles fully solve tumor delivery barriers, but because they may enable more controlled localization, combination delivery, and amplification of ferroptosis-relevant stress within resistant tumor regions [36,70,72,73,74,79,96,97]. Ferroptosis-oriented nanoparticles, ROS-generating systems, iron-based platforms, glutathione-depleting strategies, and autophagy-targeting approaches all offer possible routes for disrupting the adaptive survival programs that support residual disease [16,70,72,73,74,75,96,97]. However, direct evidence demonstrating selective eradication of PGCCs through ferroptosis-oriented nanomedicine remains limited [18,71,72,73,74,75,86,87,88,99]. 

Therefore, PGCC-directed ferroptosis nanomedicine should be viewed as a promising but still emerging framework [7,70,71,97]. Its value lies in connecting fields that are often discussed separately: therapy tolerance, PGCC biology, cancer stem-like plasticity, ferroptosis, and nanotechnology. By integrating these concepts, future research may identify more effective strategies for targeting residual therapy-tolerant populations and reducing recurrence after conventional cancer therapy.

## Figures and Tables

**Table 1:** Representative recent studies and reviews relevant to PGCC biology, therapy resistance, ferroptosis, and nanomedicine, and their limitations/potential relevant to the main focus of this present review.

Study Ref.	Approach	Key Finding	Limitation/Potential
[14]	Zoledronic acid targeting chemotherapy-resistant PGCCs	Direct evidence that a clinically used agent can reduce PGCC populations and alter PGCC-associated metabolic features.	Does not evaluate ferroptosis-oriented nanomedicine or provide an integrated PGCC–ferroptosis delivery framework.
[15]	PEGylated polymeric nanoparticles containing CIP2b in treatment-resistant endometrial cancer	Demonstrates improved tumor accumulation and enhanced efficacy through nanoparticle-mediated delivery.	Focuses on chemo-resistant tumor delivery rather than PGCC-specific biology or ferroptosis.
[16]	Nanoparticle-mediated ferroptosis strategies	Reviews nanoparticle approaches that induce ferroptosis through iron, ROS, GSH/GPX4 disruption, and combination therapy.	Does not focus specifically on PGCCs or therapy-tolerant polyploid states.
[17]	Mechanisms and toxicology of nanoparticle-induced ferroptosis	Highlights efficacy, biosafety, biodistribution, and off target ferroptosis concerns.	Addresses ferroptosis nanomedicine broadly rather than PGCC-associated resistance.
[18]	PGCC mechanisms, signaling pathways, and therapeutic strategies	Provides a broad overview of PGCC biology and potential targeting approaches.	Does not develop ferroptosis-oriented nanomedicine as a specific PGCC-targeting framework.
[19]	Radiation-induced PGCC mechanical dormancy	Demonstrates mechanically dormant, therapy-resistant PGCCs with suppressed proliferation but retained invasive features.	Establishes a new PGCC resistance phenotype but does not test ferroptosis or nanoparticle-mediated eradication.
[13]	Nanomedicine targeting cancer stem cells through HIF–Warburg–immune-evasion pathways	Integrates CSC metabolism, hypoxia, immune evasion, and targeted nanomedicine.	Focuses on CSCs rather than PGCCs and does not specifically address PGCC ferroptosis vulnerability.
[20]	Bioactive nanomedicine for multidrug-resistant colorectal cancer	Links resistance mechanisms to specific nanomedicine delivery functions and translational barriers.	Focuses on colorectal MDR rather than PGCCs as therapy-tolerant states.
Present Review	PGCCs as therapy-tolerant states; redox, lipid, iron, autophagic and metabolic vulnerabilities; ferroptosis-oriented nanomedicine	Integrates PGCC biology with therapy tolerance, ferroptosis susceptibility, and nanomedicine delivery principles.	Proposes a unified, testable framework for targeting PGCC-associated residual disease through ferroptosis-oriented nanomedicine while explicitly distinguishing established evidence from PGCC-specific hypotheses.

Table 1 Notes: PGCC: Polyploid Giant Cancer Cell, ROS: Reactive Oxygen Species, GSH: Glutathione, GPX4: Glutathione Peroxidase 4, CSC: Cancer Stem Cell, TME: Tumor, Microenvironment, MDR: Multidrug Resistance.

**Table 2:** Representative therapeutic and microenvironmental stressors and the adaptive survival responses associated with residual disease formation. These examples illustrate how conventional therapies may function not only as cytotoxic agents, but also as selective pressures that enrich for stress-adapted populations capable of persistence.

Therapeutic or Microenvironmental Stress	Primary Cytotoxic Mechanism	Adaptive Survival Response	Potential Outcomes	Key References
Taxanes (e.g., paclitaxel, docetaxel)	Mitotic arrest/mitotic catastrophe	Mitotic slippage, polyploidization	PGCC formation, persistence	[18,24,41,42,43,44,45]
Platinum Agents (e.g., cisplatin, carboplatin, oxaliplatin)	DNA crosslinking and replication stress	DNA repair activation, dormancy	Residual disease survival	[23,47,48,49,50,51,52]
Anthracyclines (e.g., doxorubicin, epirubicin)	DNA damage and ROS generation	Antioxidant adaptation, metabolic reprogramming	Oxidative stress tolerance	[18,38,53,60]
Vinca Alkaloids (e.g., vincristine, vinblastine, vinorelbine)	Microtubule depolymerization and mitotic spindle disruption	Mitotic slippage, spindle-checkpoint escape, polyploidization	PGCC formation, persistence	[42,45,63]
Antimetabolites (e.g., 5-fluororacil, gemcitabine, cytarabine)	Inhibition of nucleotide/DNA synthesis and replication stress	Cell-cycle arrest, replication-stress adaptation, polyploidization	PGCC formation, residual disease survival	[64,66]
Topoisomerase II Inhibitors (e.g., etoposide)	Topoisomerase II inhibition, DNA double-strand breaks	DNA-damage response, senescence-like arrest, endoreplication/polyploidization	Persistent polyploid cells, proliferative recovery and therapy resistance	[65,66]
Radiation	ROS-mediated oxidative injury and DNA damage	Redox buffering, stress response activation	Therapy persistence	[7,18,57,61,62]
Hypoxia/Nutrient Deprivation	Metabolic Stress	Cellular plasticity, quiescence, autophagy	Stress-adapted survival states	[7,21,23,30,39,67]

Table 2 Notes: PGCC: Polyploid Giant Cancer Cells, ROS: Reactive Oxygen Species.

**Table 3:** Representative nanoparticle platforms, therapeutic cargo, and delivery approaches relevant to resistant cancer populations.

Type of Nanoparticle	Drug Load/Cargo	Delivery Approach	Key References
Lipid Nanoparticles (LNP)	siRNA against KSP and *VEGFA*	Systemic nucleic-acid delivery and intracellular gene silencing	[79]
Lipid Nanoparticles	shRNA against *STMN1*	Protection and intracellular delivery of gene-silencing cargo	[79]
Polymeric Nanoparticles	CIP2b	PEGylated delivery to prolong circulation and increase tumor accumulation; combined with paclitaxel	[15]
Polymeric NP/micelle	Hydrophobic chemotherapeutic payloads such as paclitaxel	Encapsulation, improved solubility, prolonged circulation, controlled release	[11]
Liposome	Doxorubicin or other chemotherapeutic cargo	Passive/EPR-based delivery; PEGylation can prolong circulation; ligand modification enables active targeting	[11]
Targeted Liposome	Doxorubicin	HER2/EGFR-directed receptor targeting and tumor-cell uptake	[79]

Table 3 Notes: LNP: Lipid Nanoparticle, NP: Nanoparticle, PEG: Polyethylene Glycol, siRNA: Small Interfering RNA, shRNA: Short Hairpin RNA, KSP: Kinesin Spindle Protein, *VEGFA*: Vascular Endothelial Growth Factor A, *STMN1:* Stathmin 1, CIP2b: Ciprofloxacin Derivative 2b.

**Table 4:** Representative evidence discussed in Section 5, Section 6 and Section 7, distinguishing direct PGCC studies from mechanistic evidence derived from other therapy-tolerant or treatment-resistant cancer models.

Study Ref.	Model/Therapeutic Target	Treatment/Stress	Key Outcome	PGCCs Directly Studied?
[86]	Breast cancer cell lines with treatment-induced PGCC populations	Taxane or Aurora kinase inhibitor exposure; RSL3 or ML162	RSL3 and ML162 reduced PGCC populations; ROS modulation influenced ferroptosis response.	Yes
[87]	Irradiation-induced PGCC model in triple-negative breast cancer	Radiation followed by IKE, RSL3, FINO2, ML162, or ML210	Ferroptosis inducers reduced irradiation-induced PGCC populations.	Yes
[93]	Ovarian and breast cancer cell lines, HGSOC organoids, and patient-derived xenografts	Sublethal olaparib ± mifepristone	Olaparib induced PGCCs; mifepristone inhibited PGCC formation and daughter-cell production; combination treatment reduced tumor growth in xenografts.	Yes
[14]	Cisplatin-induced PGCC populations	Zoledronic acid	Reduced PGCC populations and altered PGCC-associated lipid accumulation, mitochondrial density, and ROS regulation.	Yes
[19]	Irradiated triple-negative breast cancer cells and xenografts	Radiation	Radiation-induced PGCCs exhibited mechanical dormancy, increased stiffness, suppressed proliferation, and retained invasion-associated features.	Yes
[59]	Drug-tolerant persister cancer cell models	GPX4 inhibition	DTP cells acquired a selective dependency on GPX4 and were vulnerable to ferroptotic death.	No
[60]	Therapy-resistant mesenchymal-like cancer cell states	GPX4 inhibition	Resistant mesenchymal-like states depended on lipid-peroxide detoxification and were highly susceptible to GPX4 inhibition.	No
[15]	Treatment-resistant type II endometrial cancer model	PEGylated CIP2b nanoparticles + paclitaxel	Improved intratumoral accumulation and circulation; combined treatment suppressed tumor progression.	No
[94,95]	Extraskeletal tumor delivery models	Zoledronic-acid nanoparticle formulations	Altered ZA biodistribution, reduced bone sequestration, improved extraskeletal tumor accumulation, and enhanced antitumor activity.	No

Table 4 Notes: DTP: Drug-Tolerant Persister; HGSOC: High-Grade Serous Ovarian Cancer; IKE: Imidazole Ketone Erastin; PGCC: Polyploid Giant Cancer Cell; ROS: Reactive Oxygen Species; ZA: Zoledronic Acid.

## Data Availability

No new data were created or analyzed in this study. Data sharing is not applicable to this article.
